# Delayed Diagnosis of Ankylosing Spondylitis: A Missed Opportunity?

**DOI:** 10.7759/cureus.5723

**Published:** 2019-09-22

**Authors:** Pooja Patel, Hira Hussain, John Fahey

**Affiliations:** 1 Rheumatology, Advocate Aurora Health, Brookfield, USA; 2 Family Medicine, St. George's University School of Medicine, Ft. Lauderdale, USA

**Keywords:** ankylosing spondylitis, stiffness, chronic low back pain, inflammatory markers, inflammatory arthritis, spondyloarthropathy, iritis

## Abstract

Ankylosing spondylitis is an inflammatory condition involving the axial spine, often associated with the human leukocyte antigen (HLA)-B27 genotype and supporting radiographic imaging findings. Patients develop symptomatic low back and/or hip pain beginning in late adolescence or early adulthood. Diagnosis of ankylosing spondylitis is based primarily on clinical presentation and imaging studies. In this article, we are presenting a case of a 40-year-old male patient who presented to the office with chief concerns of chronic mid-thoracic back pain and restricted range of motion of his neck. The imaging study obtained was suggestive of fusion of the sacroiliac joints. This article also highlights the presence of elevated inflammatory markers in the setting of the patients chronic symptomatic complaints which could have guided in early diagnosis.

## Introduction

Ankylosing spondylitis is a chronic inflammatory disease, affecting the axial skeleton, entheses, and peripheral joints. Ankylosing spondylitis (AS) is categorized under axial spondyloarthropathy and is differentiated from non-axial spondyloarthropathy due to the significant involvement of the sacroiliac joints observed in AS. The age of onset of AS is typically in late teens and early 20’s. The incidence rate of AS is more common among men [1]. The estimated prevalence of AS in various countries ranges from 0.7 to 49 per 10,000 [2]. Hereditary prevalence of AS is high especially amongst siblings with positive human leukocyte antigen (HLA)-B27 [3].

## Case presentation

A 40-years-old male patient with past medical history consistent of congenital left leg-length discrepancy of about three-fourth inches, status postoperative multiple corrective surgeries for the same, severe bilateral hip osteoarthritis with status postoperative right hip resurfacing surgery, presents to the office with chief concerns of chronic thoracic back pain, and restricted range of motion of his neck. The patient reports intermittent back pain since he was in his 20’s. His neck pain started approximately five to seven years ago. The patient rates his pain an average of three on a pain scale of 10; ranging between one to eight out of 10. He is unable to bend down to pick up objects. His neck pain and stiffness are markedly limiting his activities. He feels his pain is muscular in nature, especially in his neck. The patient conveys his inability of touching his toes during his high school. The patient’s stiffness lasts all day; hot shower in the morning helps decrease it minimally. He reports progressively worsening difficulty sleeping. Until four years ago, he was able to practice yoga which significantly helped his pain. The patient tried various nonsteroidal anti-inflammatory agents (NSAID’s) without significant improvement. Muscle relaxers caused increased drowsiness and weakness. Massage therapy to his neck markedly alleviates his neck pain. Physical therapy and chiropractic services were unimpressive in the past. The patient denies any bowel/bladder incontinence, saddle anesthesia, fever, shortness of breath, bleeding per rectum, tingling or numbness. He denies any recent trauma or injury. The patient denies any gastrointestinal symptoms (irritable bowel syndrome, Crohn’s disease or ulcerative colitis), psoriasis or inflammation of his eyes (iritis, scleritis or episcleritis.) The patient has not had his human leukocyte antigen (HLA)-B27 marker checked. Otherwise, the patient reports to be in good health. The patient states people have told him that he walks like his father. His father had difficulty walking with his head up due to neck stiffness and used a back brace for support. The patient’s brother has Crohn’s disease. He is unaware of his brother’s HLA-B27 marker status. The patient has two healthy children: a son and a daughter. The patient reports drinking about one alcoholic beverage a day ranging from beer, rum and coke or a martini. He smokes a cigar once a month.

Upon examination, his vitals are within normal limits. The patient is conscious, cooperative, well-oriented in time, place and person. The patient has diaphragmatic respiration with lungs clear on auscultation. Cardiovascular examination is normal. The abdomen is soft, nontender without organomegaly. His extremity examination is consistent with left leg-length discrepancy of about three-fourth inches, left leg shorter than the right. The left leg is markedly atrophic and weaker compared to the right leg. No range of motion of his left ankle joint. Left foot deformity present. The patient has only five percent range of motion of his neck. On neurological examination, he walks with a stiff posture, limping gait without touching his left heel to the floor. On noticing his left foot while walking, he first walks on his toes and then supports himself on his left heel. He cannot bend down to touch his toes.

**Figure 1:**
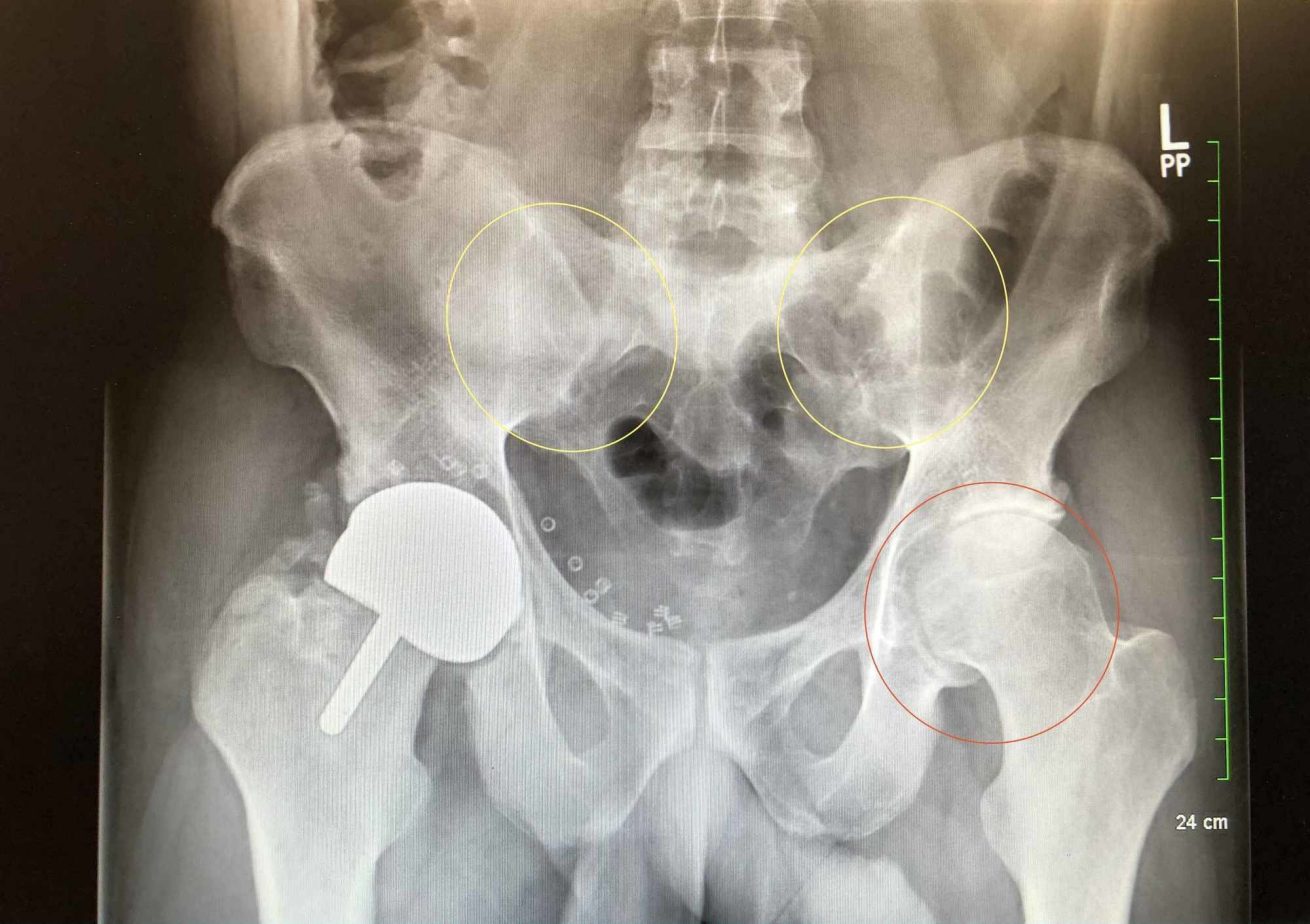
X-ray Image of the sacroiliac spine Findings: There appears to be a fusion of the sacroiliac (SI) joints (yellow circles). There is a marked narrowing of the left hip joint (red circle). There is a hip resurfacing prosthesis on the right. Impression: There is a fusion of the SI joints. Prominent degenerative change and narrowing of the left hip joint. Resurfacing prosthesis on the right hip.

**Figure 2:**
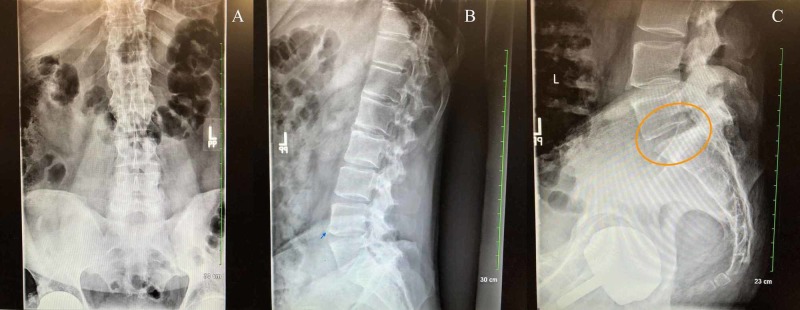
X-ray of the lumbar spine Findings: The heights of the lumbar vertebral bodies are normal. There are degenerative facet changes at lumbar spinal (L) levels 4 and 5 (L4-5). Mild narrowing of lumbar spine level 5 and sacral disc 1 space (L5-S1) (image C, orange circle). There is some anterior spurring of L4-L5 (image B, blue arrow) and at L1-L2. Remaining lumbar disks are well preserved. Impression: Degenerative changes as described above. Findings are most marked in the facets at L4 and L5. There is no evidence of vertebral fracture.

**Figure 3:**
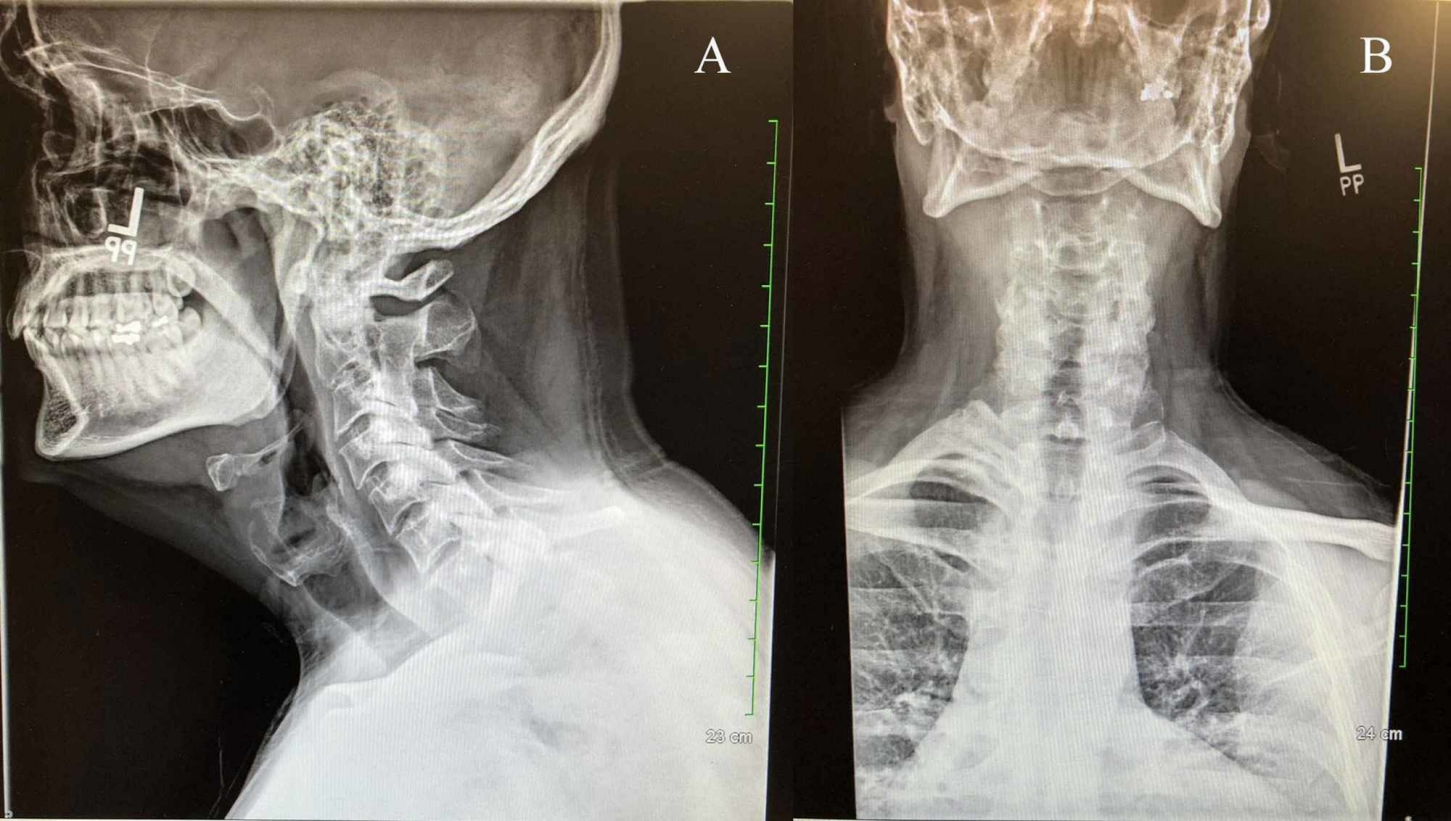
X-ray of the cervical spine Findings: The heights of the cervical vertebral bodies are normal. There is the mild narrowing of the cervical C2-C3 disc space. There is a mild anterior spurring at the cervical C3-C4 disc levels. There does not appear to be significant narrowing. Remaining cervical disc spaces are fairly well-preserved. There are mild degenerative facet changes in the mid and upper cervical spine. The odontoid appears intact. Impression: Mild degenerative disc changes. Degenerative facet changes. There is no evidence of cervical spine fracture.

The patient’s previous and recent laboratory test results and imaging results were reviewed with him. His complete blood count, comprehensive metabolic panel and thyroid-stimulating hormone levels were normal (Table 1; Figures 1, 2, 3).

**Table 1:** Rheumatologic serology laboratory test results NA - not applicable; units/mL - units per milliliter; mg/dL - milligram per deciliter; mm/hour - millimeter per hour

Component	Normal reference range	Eight years ago	Six years ago	Five years ago	Two years ago	Recent
Erythrocyte sedimentation rate	0-20 mm/hour	22	20	24	44	26
C-reactive protein	< 1.0 mg/dL	NA	3.1	2.6	3.3	NA
Cyclic citrulline peptide antibody	<20 units	2	NA	NA	3	NA
Rheumatoid factor	<15 Units/mL	<20	NA	NA	<10	NA
Antinuclear antibody screen	Negative	Negative	NA	NA	NA	NA

On assessment, the patient’s symptoms are suggestive of ankylosing spondylitis (AS). The constellation of the limited range of motion of his neck, lumbar spine stiffness, elevated inflammatory markers, and fused sacroiliac joints (SI joints) confirmed on an imaging study, are supportive of ankylosing spondylitis as the diagnosis. Furthermore, his family history consistent with his father having similar symptoms and posture, and his brother with Crohn’s disease, increases the patient’s probability of spondyloarthropathy. We discussed ankylosing spondylitis in detail with the patient, including the extra-articular symptoms associated with AS. Due to the SI joint, and the absence of confluent ossification of at least four continuous vertebral levels makes diffuse idiopathic skeletal hyperostosis (DISH) unlikely. The patient did not present with active inflammation or synovitis of his peripheral joints (hands, knees or wrists), along with negative rheumatoid factor and cyclic citrulline peptide antibody levels, making inflammatory arthropathy like rheumatoid arthritis less likely. After discussing the high pretest probability of AS with a positive human leukocyte antigen (HLA)-B27 marker, and its hereditary nature, a laboratory test checking for HLA-B27 marker was ordered.

For the patient’s management, the importance of good posture, ergonomics, and core-strengthening exercises was reiterated. The patient was encouraged to lose weight, quit smoking and limit his alcohol consumption. He was recommended undergoing physical therapy to help with his neck pain. Unfortunately, due to the progression of his medical condition, the patient was made aware of the irreversible complications. To avoid further progression, we recommended initiating subcutaneous citrate-free adalimumab injection 40 milligrams per 0.4 milliliters once every 14 days. We prescribed him a muscle relaxer, tizanidine 2-milligram tablet, to take at bedtime to help his muscle spasm. It was recommended taking occasional oral NSAID’s as needed for pain. 

The patient’s HLA-B27 marker, QuantiFERON® tuberculosis test, and hepatitis B surface antigen were negative. The patient followed-up at the office about three months later, reporting a gradual improvement in his neck pain. The patient did develop an episode of iritis in the interim; he was prescribed prednisolone topical eye drops by his ophthalmologist. The patient is currently in stable condition and continues to follow-up with his team of providers. 

## Discussion

Ankylosing spondylitis (AS), an axial spondyloarthropathy is often characterized by a fusion of the spine. It is a form of arthritis which mostly affects the spine but can also involve other joints. Inflammation of the spinal joints leads to severe, chronic pain. As the inflammation progresses, it can lead to ankylosis which is new bone formation in the spine. This causes sections of the spine to become fused. There is a strong association with HLA-B27. This is a normal gene found in eight percent of the Caucasian population. Testing for this gene is not diagnostic for AS due to the variations seen amongst different ethnicities. However, in the Caucasian race, it is a strong predictor [4].

Patients complain of developing symptoms before the age of 40 years. Patients will usually present with fatigue, low-grade fever, weight loss, lower back pain and stiffness along with limitations in movement. They may also present with enthesitis which is inflammation at the site where tendons insert into bone [1]. The healing that occurs following entheses may eventually lead to scarring of the tissue. This may result in the formation of more bone. This will lead to an increase in spinal fractures due to the restricted range of movement. Affected individuals may develop a forward curvature of the spine, also known as kyphosis, due to the way the spine is fusing. Lower back pain and stiffness are worse with rest and improve with physical activities [5]. As more of the spine is affected, it becomes brittle and more prone to fractures. Patients may also present with chest pain that may feel like a heart attack or will complain of pain while taking deep breaths. This is due to long term inflammation of the joints between the ribs and the spine along with the joints between the ribs and sternum. The inflammation will eventually end up with long term scarring resulting in decreased chest expansion. The extra-articular manifestations of AS are summarized in (Table 2) [6].

**Table 2:** Extra-articular manifestations of ankylosing spondylitis

System Involved	Presentation
Musculoskeletal:	
Axial Involvement	Axial involvement predominates; initially is symmetric, involving the sacroiliac joints and lower spine, progressing cranially; does not skip regions
Peripheral Involvement	Peripheral involvement consists of enthesitis; may have asymmetric large joint oligoarthritis, including hips and shoulders; hip involvement causes significant functional limitations; dactylitis is uncommon
Dermatologic:	Skin findings are not characteristic; psoriatic like lesions may occasionally occur
Ophthalmologic:	Anterior uveitis
Gastrointestinal:	Asymptomatic intestinal ulcerations
Genitourinary:	Urethritis
Cardiovascular:	Aortic valve disease; aortitis; conduction abnormalities; coronary artery disease
Pulmonary	Restrictive lung disease from costovertebral rigidity; apical fibrosis
Bone Quality	Falsely elevated bone mineral density from syndesmophytes; increased risk of spine fracture

Ankylosing spondylitis can be diagnosed via a thorough physical exam, imaging studies of the lumbar and pelvis, family history of AS, along with laboratory tests inclusive of inflammatory markers (elevated C-reactive protein and erythrocyte sedimentation rate) and to evaluate for HLA-B27 marker. If the radiographic imaging studies are inconclusive, computer tomographic study of the sacroiliac joints or magnetic resonance imaging of the sacroiliac joints should be obtained. The differential diagnosis is summarized in (Table 3) [7].

**Table 3:** Differential diagnosis of ankylosing spondylitis N/A - not applicable

Diagnosis:	Clinical presentation:	Investigations:	Key points for ankylosing spondylitis:
Lumbar strain or muscle spasm	Acute onset often with precipitating event.	N/A	Often reports a history of chronic skeletal pain.
Herniated disc	Acute onset with pain radiating below the knee, often associated with neurological deficits (numbness, tingling, weakness, or absent or decreased deep tendon reflexes).	Magnetic resonance imaging of the lumbar spine.	Reports a history of chronic arthropathy.
Osteoarthritis	Pain is aggravated with activity, and often has an absence of inflammatory symptoms.	Radiographic evaluation suggestive of asymmetric joint-space narrowing, subchondral sclerosis, osteophytes, and bony cysts.	Ankylosing spondylitis pain improves with movement and has an association with inflammatory symptoms.
Rheumatoid arthritis	Predominantly involves multiple, small, peripheral joints of the hands and feet. Usually spares the sacroiliac joints with little effect on the rest of the spine except for cervical spines 1 and 2.	Elevated inflammatory markers, positive rheumatoid factor and cyclic citrulline Peptide antibody. Occasionally, positive antinuclear antibody screen.	Predominately involves the sacroiliac joints.
Psoriatic arthritis	Involves multiple peripheral joints, in addition to erythematous sharply defined plaques.	Clinical diagnosis	Ankylosing spondylitis may occasionally present with concomitant psoriasis.
Reactive arthritis (formerly known as Reiter's syndrome)	Usually presents with symptoms of sporadic arthritis, urethritis and/or conjunctivitis.	Complete blood count, liver panel, urinalysis, urine culture and sensitivity, nucleic acid amplification assay test for chlamydia and gonorrhea, antinuclear antibody screen, human leukocyte antigen B27, parvovirus antibody screen.	Ankylosing spondylitis symptoms are insidious onset, characterized with history of chronic pain.
Irritable bowel disease	Reports a history of abdominal pain/tenderness, diarrhea, bleeding per rectum, malaise, fatigue, arthralgia, iritis, uveitis.	Inflammatory markers, stool examination for bacterial culture, ova and parasite, Clostridium difficile toxins, computed tomographic study of the abdomen and pelvis, colonoscopy.	Ankylosing spondylitis may or may not present with symptoms of irritable bowel disease.

Treatment involves supportive and symptomatic management. Physical therapy is vital in preventing postural changes and maintaining a good range of motion. Non-steroidal anti-inflammatory drugs (NSAIDS) and anti-tumor necrosis factor medications are used for symptomatic relief. Surgery is the last resort in those with advanced disease. The following table summarizes the medical management of patients with AS (Table 4) [8, 9].

**Table 4:** Medical management of ankylosing spondylitis

Treatment	Recommendation	Note
Non-steroidal anti-inflammatory drugs	Celecoxib (Celebrex®) 100 to 200 milligrams once or twice a day; diclofenac (Voltaren®) 25 to 50 milligrams, two to four times a day; etodolac (Lodine®) 200 to 500 milligrams twice a day; ibuprofen (Motrin®, Advil®) 400 to 800 milligram, three to four times a day; indomethacin (Indocin®) 25 to 50 milligrams, three to four times a day; meloxicam (Mobic®) 7.5 to 15 milligrams, once or twice a day; nabumetone (Relafen®) 500 to 750 milligrams, once a day; naproxen (Aleve®) 220 milligrams twice a day; naproxen (Naprosyn®) 250 to 500 milligrams twice a day; piroxicam (Feldene®) 10 to 20 milligrams once a day; sulindac (Clinoril®) 150 to 200 milligrams one to two times a day.	All non-steroidal anti-inflammatory drugs have the potential to cause upset stomach, peptic ulcer disease, internal bleeding, heart damage, and kidney damage.
Tumor necrosis factor inhibitors	Etanercept 50 milligrams per 0.5-milliliter injection once a week subcutaneously; adalimumab 40 milligrams per 40-milliliter injection every other week subcutaneously; infliximab 5 milligrams per kilogram every 6-8 weeks intravenously; golimumab 50-milligram injection once a month subcutaneously; certolizumab 200 milligrams injection every other week subcutaneously.	Too much tumor necrosis factor leads to inflammation. Levels of this substance are increased in autoimmune diseases like ankylosing spondylitis. Tumor necrosis factor inhibitors are antibodies made from human or animal tissue to keep tumor necrosis factor levels steady. Patients are at increased risk of infection, lymphoma, or tuberculosis. Recommend screening patients with a skin test and a chest x-ray before beginning therapy.
Disease-modifying anti-rheumatic drugs	Sulfasalazine 1000 milligrams twice a day orally; secukinumab (Cosentyx®) with a loading dose of 150 mg at weeks 0, 1, 2, 3 and 4 and then every four weeks thereafter; methotrexate once a week.	Sulfasalazine may cause yellow/orange urine or skin. Secukinumab - human Immunoglobulin G1k monoclonal antibody that binds to interleukin 17A. Methotrexate may cause swollen gums or mouth sores.

Some complications of ankylosing spondylitis include restrictive lung disease due to thoracic spine involvement, eye involvement, cauda equina syndrome, spine fractures with spinal cord injury, osteoporosis and spondylodiscitis [10-13].

Majority of patients with ankylosing spondylitis have persistent symptoms for decades varying from unremitting spinal pain, hip destruction, and spinal fusion. Depending on the disease severity, about 10-30 percent of patients develop working disability after 10 years. A number of prognostic indicators have been identified (Table 5) [14].

**Table 5:** Prognostic indicators in patients with ankylosing spondylitis

Prognostic indicators in patients with ankylosing spondylitis:
Hip arthritis
Sausage-like fingers or toes
High erythrocyte sedimentation rate (>30 millimeter per hour)
Limitation in range of motion of the lumbar spine
Oligoarthritis
Onset less than 16 years of age

## Conclusions

Ankylosing spondylitis is an inflammatory axial spondyloarthropathy consistent with chronic symptomatic complaints. The incidence rate of the disease is common amongst younger individuals, complaining of intermittent chronic arthralgia. A thorough history of presenting illness including a family history of any back pathologies or any associated-symptoms of spondyloarthropathy should be enquired. The presence of elevated inflammatory markers and imaging studies evaluating for sacroiliitis should be obtained in patients suspected to have AS. Physical therapy and exercise should be encouraged in patients with ankylosing spondylitis to maintain a good range of motion. Pertinent to our patient, literature review on ankylosing spondylitis suggests that early diagnosis and appropriate therapy is known to slow the progression of the disease. 
